# The Gut Microbiota during a Behavioral Weight Loss Intervention

**DOI:** 10.3390/nu13093248

**Published:** 2021-09-18

**Authors:** Maggie A. Stanislawski, Daniel N. Frank, Sarah J. Borengasser, Danielle M. Ostendorf, Diana Ir, Purevsuren Jambal, Kristen Bing, Liza Wayland, Janet C. Siebert, Daniel H. Bessesen, Paul S. MacLean, Edward L. Melanson, Victoria A. Catenacci

**Affiliations:** 1 School of Medicine, University of Colorado Anschutz Medical Campus, Aurora, CO 80045, USA; Daniel.Frank@cuanschutz.edu (D.N.F.); Sarah.Borengasser@cuanschutz.edu (S.J.B.); Danielle.Ostendorf@cuanschutz.edu (D.M.O.); Diana.Ir@cuanschutz.edu (D.I.); Puujee.Jambal@cuanschutz.edu (P.J.); Kristen.Bing@cuanschutz.edu (K.B.); Liza.Wayland@cuanschutz.edu (L.W.); jsiebert@cytoanalytics.com (J.C.S.); Daniel.Bessensen@cuanschutz.edu (D.H.B.); Paul.Maclean@cuanschutz.edu (P.S.M.); Ed.Melanson@cuanschutz.edu (E.L.M.); Vicki.Catenacci@cuanschutz.edu (V.A.C.); 2 Eastern Colorado Veterans Affairs Geriatric Research, Education and Clinical Center, Denver, CO 80045, USA

**Keywords:** microbiota, weight loss, intermittent fasting, obesity

## Abstract

Altered gut microbiota has been linked to obesity and may influence weight loss. We are conducting an ongoing weight loss trial, comparing daily caloric restriction (DCR) to intermittent fasting (IMF) in adults who are overweight or obese. We report here an ancillary study of the gut microbiota and selected obesity-related parameters at the baseline and after the first three months of interventions. During this time, participants experienced significant improvements in clinical health measures, along with altered composition and diversity of fecal microbiota. We observed significant associations between the gut microbiota features and clinical measures, including weight and waist circumference, as well as changes in these clinical measures over time. Analysis by intervention group found between-group differences in the relative abundance of *Akkermansia* in response to the interventions. Our results provide insight into the impact of baseline gut microbiota on weight loss responsiveness as well as the early effects of DCR and IMF on gut microbiota.

## 1. Introduction

Nearly 40% of U.S. adults are afflicted with obesity, a condition that accounts for USD 147 billion in annual healthcare costs [1]. Obesity increases risk for numerous chronic diseases, including cardiovascular disease and type 2 diabetes [1]. Even modest (5–10%) weight loss facilitates significant improvements in cardiovascular and metabolic function [2]. Lifestyle interventions that focus on dietary energy restriction and increasing physical activity are the cornerstone of obesity treatment [3]; however, there is substantial inter-individual variability in weight loss with current lifestyle approaches [4,5]. A greater understanding of factors that impact individual variability in the weight loss response may pave the way towards more effective, personalized interventions [6].

The gut microbiota appear to play an important role in the development of obesity [7,8,9,10,11], and may also significantly contribute towards this variability in weight loss responsiveness [12,13]. Alterations in the composition or diversity of gut microbiota (i.e., dysbiosis) have been linked to the pathogenesis of obesity in both animal models and humans [14,15] through mechanisms involving energy balance/nutrient absorption, inflammatory pathways, appetite regulation and/or the generation of small molecules that alter the metabolism [16]. Weight loss has been shown to produce changes in the gut microbiota [17], and there is evidence that gut microbiota and gut microbiota–derived metabolites may be important mediators of the response to dietary energy restriction [18,19]. For example, a more diverse baseline gut microbiota has been associated with enhanced response to a dietary weight loss intervention [20].

In order to assess the potential role of the gut microbiota in weight loss, we collected stool samples from participants in two recruitment cohorts of an ongoing randomized behavioral weight loss intervention trial called DRIFT2 (Daily caloric Restriction versus Intermittent Fasting Trial 2). The goal of DRIFT2 is to compare weight loss produced by intermittent fasting (IMF, 80% restriction of energy intake on three non-consecutive days per week with no restriction of intake on intervening days) to the current standard of care dietary weight loss approach of daily caloric restriction (DCR). In this study, both IMF and DCR groups target an equivalent weekly dietary energy deficit (34%), receive identical exercise prescriptions, and receive a comprehensive group-based behavioral weight loss program. In this ancillary study, we report the results of 16S rRNA gene profiling of fecal microbiota among 59 individuals (*n* = 34 IMF and *n* = 25 DCR) at baseline and three months into the intervention, the period when adherence was likely highest [21]. We examine how clinical measures and the gut microbiota change in response to the first three months of the lifestyle weight loss intervention and assess the cross-sectional and longitudinal relationships between the clinical measures and the gut microbiota. We focus primarily on two key clinical health measures of response to a weight loss intervention: weight and waist circumference. Weight loss is the primary endpoint of the parent trial, and waist circumference is a critical indicator of overall cardiometabolic health [22]. Since the trial is ongoing and the study investigators of the parent trial are blinded to outcome measures by randomized group assignment, we do not report differences in clinical measures (weight, waist circumference) by intervention group. We focus most of our analyses on the overall cohort, and then we assess whether there are differences in our findings among the participants undergoing DCR versus IMF dietary interventions, acknowledging that interpretation of any between-group differences in the gut microbiota is limited without concomitant knowledge of whether there are differences between the groups in changes in clinical measures. Taken together, our results offer insight into the role of the gut microbiota in these weight loss interventions.

## 2. Materials and Methods

**Figure 1:**
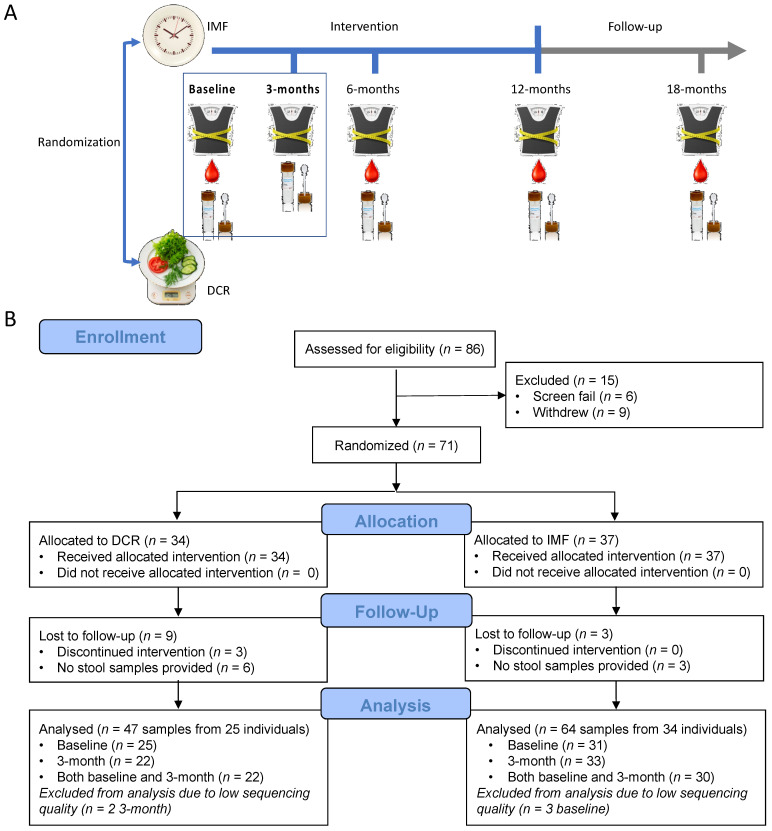
Study design and CONSORT Diagram for DRIFT2 and this ancillary study. (**A**) DRIFT2 is a 12-month behavioral weight loss intervention of daily caloric restriction (DCR) versus intermittent fasting (IMF) with data collections involving anthropometry, blood and stool collections at the time increments shown. There is also a follow-up data collection six-months post-intervention. This ancillary study focuses on measures collected at baseline and three months. (**B**) The DRIFT2 study assessed 86 individuals and randomized 71 (34 DCR and 37 IMF) in Cohorts 1 and 2. There were nine individuals from DCR and three from IMF lost to follow-up. The gut microbiome analyses involved 25 individuals randomized to DCR and 34 to IMF.

This study is ancillary to an ongoing 12 month behavioral-based weight loss trial, DRIFT2 (ClinicalTrials.gov NCT03411356; registered 26 January 2018), at the University of Colorado Anschutz Medical Campus. The flow diagram of the study enrollment, allocation, follow up, data collection and analysis is shown in Figure 1. This study was approved by the Colorado Multiple Institutional Review Board and is conducted at the University of Colorado Anschutz Health and Wellness Center (CU-AHWC). All research is performed in accordance with relevant guidelines/regulations.

### 2.1. Participants and Intervention

This ancillary study included men and women who are overweight or obese (*n* = 59 participants, DCR = 25, IMF = 34), participating in the parent pragmatic trial during the initial 2 of 5 planned cohorts (starting 8 April 2018 and 13 February 2019). The data for this ancillary study includes information collected at baseline and three months into the intervention for the participants who provided a fecal sample at one of these study visits. As described previously [23], all participants in the parent trial receive a 12 month comprehensive behavioral weight loss intervention involving an energy-restricted diet, increased physical activity, and group-based behavioral support. Participants are randomized to focus on either IMF or DCR as the dietary strategy during the 12 month intervention. Primary inclusion criteria for the parent trial included age of 18–55 years, BMI of 27–45 kg/m^2^, sedentary (defined as self-report of < 150 min/week of voluntary exercise at moderate or greater intensity over past three months), and live or work within 30 min of CU-AHWC. Primary exclusion criteria for the parent trial included cardiovascular disease, diabetes, uncontrolled hypertension, uncontrolled thyroid disease, current nicotine use, use of medications known to affect body weight, weight change > 5% in the past three months, major psychiatric disorder, eating disorder, current moderate to severe depression, and current alcohol or substance abuse. Women who were pregnant, lactating, or planning to become pregnant in the next 12 months were also excluded. After providing written informed consent and undergoing study screening procedures, the participants were assigned to treatment conditions (1:1), using an a priori randomization list generated by the study statistician and blinded to principal investigators. The randomization list was generated, using block randomization with a block size of 4 and stratified by the gender of participants. The study statistician witnessed the process of assigning treatment run by research assistants.

### 2.2. Dietary Prescription

Weight maintenance energy requirements were determined for all participants as the measured resting metabolic rate multiplied by an activity factor of 1.5. Participants randomized to DCR were given a calorie goal designed to produce a ~34% daily energy deficit from baseline estimated individual weight maintenance energy requirements, which is a standard dietary approach to weight loss recommended in current obesity treatment guidelines [3]. Participants randomized to IMF were given a calorie goal designed to limit energy intake to 20% of the estimated baseline individual daily weight maintenance energy requirements on three non-consecutive days per week and were instructed not to restrict energy intake on intervening days (but they were encouraged to make healthy food and portion choices). This strategy should result in a ~34% weekly energy deficit from baseline estimated individual weight maintenance energy requirements in the IMF group. It is important to note that since this is an ongoing trial, we are not reporting between-group differences in clinical measures during the intervention by randomized group assignment. 

### 2.3. Physical Activity Prescription

All participants were provided access to the CU-AHWC fitness center and received a recommendation to gradually increase their moderate intensity physical activity levels to 300 min/week over the initial 6 months. This target is consistent with current physical activity guidelines for weight management [24,25]. Behavioral support for physical activity was provided within the weight loss program.

### 2.4. Behavioral Support 

Behavioral support involved group meetings (60–75 min) led by a registered dietician (RD). The intervention groups met separately. Group meetings were held weekly during the initial three months of the intervention, and then every two weeks for the remainder of the 12 month intervention. Attendance at group sessions was tracked and body weight (for participant feedback only) was assessed at weekly behavioral sessions. The curriculum for DCR was based on the Colorado Weigh behavioral weight loss program, which uses a skills-based approach and cognitive behavioral strategies for lifestyle modification with a dietary focus on DCR [26,27]. The curriculum uses a mix of large group discussion, small breakout discussions, visual demonstrations, and written exercises. Topics covered include the following: realistic weight loss goal setting, calorie counting, portion control, self-monitoring strategies including self-weighing and keeping food logs, mindful eating, stress management, cognitive restructuring, improving personal food environments and social networks, and strategies to overcome barriers to healthy eating. DCR participants were guided to track daily caloric intake by keeping a daily food log and weighing and measuring all foods. The RD reviewed all food logs and provided individualized written feedback. The IMF curriculum involved comparable weekly themes to DCR with modifications to focus on behavioral support specific to IMF. IMF participants were guided to track caloric intake on fast days only by keeping a fast day food log and weighing and measuring all foods. The RD reviewed all food logs and provided individualized written feedback.

### 2.5. Data Collection

#### 2.5.1. Anthropometric Measurements 

All measures were collected by trained study personnel blinded to the intervention group. Body weight was measured, using a digital scale accurate to ±0.1 kg at baseline and three months. Height was measured to the nearest 1 mm with a stadiometer at baseline. Waist circumference was measured at baseline and three months with a tape measure just over the iliac crest. 

#### 2.5.2. Dietary Intake

Patterns of energy intake on fed and fast days were collected at baseline and three months, using seven-day diet records, which were analyzed by Colorado Clinical and Translational Sciences Institute Nutrition Core personnel blinded to the study group assignment, using Nutrition Data System for Research software (University of Minnesota, Minneapolis, MN, U.S.A.). Energy intake and percentage intake of total carbohydrates, protein and fat were used in our analyses.

#### 2.5.3. Physical Activity

Participants were asked to wear the activPAL device (activPAL v4, PAL Technologies Ltd., Glasgow, U.K.) continuously for seven days in order to assess physical activity patterns. The device was waterproofed with a nitrile sleeve before attaching it onto the participant’s anterior thigh, using a Tegaderm transparent film. Data recorded on the device were processed using the CREA algorithm from the activPAL’s data processing software, PALbatch (version 8.10.09.43, PAL Technologies Ltd., Glasgow, U.K.). We used the most conservative PALbatch setting, the 24-h protocol, which allows for four hours of non-wear time for each 24 h day. Data were considered valid and were used for analysis if the device was worn for > 20 h/d on ≥ 4 days (including ≥ 1 weekend day). Average time (min/day) spent stepping at a cadence of 75 steps/min and average time (min/day) spent sitting were used in our physical activity analyses. The activPAL device has been shown to be reliable and valid in estimating physical activity and sedentary behavior in adults [28] with high accuracy in estimating different activity intensity categories [29].

#### 2.5.4. Blood Collection

A 12-h fasting whole blood sample was obtained at baseline by a trained phlebotomist and immediately processed for plasma, which was stored at −80 °C for future analyses. Plasma was used for assessment of glucose, HDL, and triglycerides by the Colorado Clinical and Translational Sciences Institute Core Laboratory, blinded to the randomized group assignment. 

#### 2.5.5. Stool Collection

Participants were asked to provide stool samples at baseline and at three months into the intervention. They were given EasySampler^®^ Stool Collection Kits (ALPCO, Salem, NH, U.S.A); they performed the stool collection at home and brought them on ice packs to their in-person visit, at which time they were frozen at −80 °C. They were asked to provide a sample from the day of their visit; in cases where this was not possible, they froze the sample in their freezer and brought it the following day to their in-person visit.

#### 2.5.6. 16S rRNA Gene Profiling

Stool samples were homogenized, using the Roche MagNA Lyser (Roche Inc, Basel, Switzerland). DNA was extracted from the homogenized fecal isolates, using the QiaAmp PowerFecal DNA kit (Hilden, Germany). Bacterial profiles were determined by broad-range amplification and sequence analysis of 16S rRNA genes, following our previously described methods [30,31]. In brief, amplicons were generated, using primers that target approximately 400 base pairs of the V3V4 variable region of the 16S rRNA gene. PCR products were normalized, using a SequalPrep^TM^ kit (Invitrogen, Carlsbad, CA, U.S.A.), pooled, lyophilized, purified and concentrated, using a DNA Clean and Concentrator Kit (Zymo, Irvine, CA, U.S.A.). Pooled amplicons were quantified, using Qubit Fluorometer 2.0 (Invitrogen, Carlsbad, CA, U.S.A.). The pool was diluted to 4nM and denatured with 0.2 N NaOH at room temperature. The denatured DNA was diluted to 15 pM and spiked with 25% of the Illumina PhiX control DNA prior to loading the sequencer. Illumina paired-end sequencing was performed on the MiSeq platform with versions v2.4 of the Miseq Control Software and of MiSeq Reporter, using a 600 cycle version 3 reagent kit.

#### 2.5.7. Microbiome Data Processing

The reads were quality filtered and trimmed to a uniform length based on the average position of first low-quality base pair among all samples, using Qiime2 2019.10 software [32]. DADA2 was run with default parameters to de-noise the data and find exact sequence counts across samples. The quality-filtered sequences were inserted into the Silva 12.8 taxonomic database [33] using SEPP [34]. Analyses were standardized at 3407 sequences per sample to maximize the inclusion of sequences and to avoid biases. 

### 2.6. Statistical Analyses

We compared cohort demographic characteristics by intervention group, using Student’s *t*-tests for continuous variables and chi-squared or Pearson’s exact tests for categorical variables. 

#### 2.6.1. Change in the Gut Microbiota over Time 

We used repeated measures permutational ANOVA of the unweighted and weighted UniFrac [35] distance metrics to assess qualitative and quantitative changes in the overall taxonomic composition over time, controlling for time, age, sex and intervention group, evaluating a time by intervention interaction, and allowing for correlation by subject [36]. We used the ANCOM (analysis of composition of microbiomes) method [37] with linear mixed models of the longitudinal data from baseline and three months to assess the gut microbiota genera that changed over this time period or that showed interactions between time and intervention group, additionally controlling for age and sex, and allowing for correlation by subject. For ANCOM, we used a prevalence cutoff of 0.9 for inclusion and a cutoff for the W statistic of 0.7. We used a multiple comparison-adjusted *p*-value of 0.1, using the Benjamini–Hochberg false discovery rate (FDR) [38], due to the small sample size. Four indices of alpha-diversity were examined (observed, evenness, Shannon diversity index and Faith’s Phylogenetic Diversity (PD)), in order to assess different aspects of alpha-diversity, such as evenness, richness and phylogenetic relatedness. Linear mixed regression models were used with alpha-diversity at baseline and three months as outcomes and time, age, sex, and intervention group as predictors with a random effect for individual. We also evaluated a time by intervention interaction. 

#### 2.6.2. Cross-Sectional Associations between Gut Microbiota Measures and Clinical Measures

We examined the cross-sectional association between demographic factors and gut microbiota beta-diversity UniFrac metrics [35] at baseline and three months, using permutational ANOVA [39]. We used similar methods, controlling for age and sex, as well as intervention group at three months, to assess the association with clinical health metrics: weight and waist circumference at baseline and three months, and additionally MetS score and its components at baseline (triglycerides, glucose, blood pressure and HDL). 

#### 2.6.3. Association between Gut Microbiota and Change in Clinical Outcomes

We examined the association between the baseline gut microbiota beta-diversity UniFrac metrics and percent change in weight and in waist circumference, using MiRKAT [40] and controlling for age and sex. We used a similar approach to assess the association between change in beta-diversity UniFrac metrics from baseline to three months (calculated using PLDist) [41]. We repeated the MIRKAT analyses (1) stratified by intervention group and (2) in the subsets of individuals with dietary information, controlling for caloric intake and macronutrient percentages, and with physical activity data, controlling for moderate cadence stepping time and sitting time (or change in these variables for the analysis of change in beta-diversity). Linear regression models were used to model percent change in weight and in waist circumference as a function of baseline alpha-diversity metrics, controlling for age, sex, and intervention group as predictors. 

#### 2.6.4. Specific Gut Microbiota Taxa Predictive of Change Clinical Outcomes

In order to identify specific gut microbiota genera predictive of percent change in weight and waist circumference, we used a feature selection technique based on random forests called VSURF (variable selection using random forests) [42]. We applied VSURF to predict the percent change in these outcomes based on baseline relative abundance of gut microbiota genera. We applied the same approach using change in abundance of gut microbiota genera [41]. Random forests allow for complex relationships among the selected predictors and between predictors and the outcome, which can be difficult to interpret. Thus, we also evaluated the linear associations between each identified predictor and the clinical outcomes using linear regressions, controlling for age, sex and intervention group and evaluating the interaction between the identified predictors and intervention group (using an interaction *p*-value cutoff of 0.1). In order to assess the performance accuracy of random forests to predict the outcomes, we used repeated 3-fold cross-validation (100 repetitions) of random forests using the following: (1) all genera that were present in at least 10% of the samples and had a minimum relative abundance across all samples greater than 0.1%; and (2) the subset of genera selected by VSURF as most highly predictive [43].

#### 2.6.5. Predictors of Changes in Gut Microbiota Beta-Diversity

We used permutational ANOVA [39] to assess whether the change in clinical measures (percent change in weight and waist circumference), diet (change in average caloric intake and in percentages of macronutrients) or physical activity (stepping time with cadence ≥ 75 and sitting time) were predictive of the change in gut microbiota beta-diversity based on UniFrac metrics [41] from baseline to three months. 

## 3. Results

### 3.1. Study Overview

This study is ancillary to an ongoing randomized lifestyle weight loss trial (DRIFT2), comparing weight loss generated by IMF to DCR over one year in healthy adults who are overweight or obese. The study design for the parent trial, DRIFT2, is depicted in Figure 1A. Individuals who are overweight or obese (age 18–55 years, BMI: 27–45 kg/m^2^) and who are otherwise healthy were randomized 1:1 to either DCR or IMF, stratified by sex. To determine whether features of the gut microbiota assessed at either baseline or in the first three months of the intervention are associated with early clinical outcomes, we performed an ancillary study (Figure 1B) of baseline and three month samples collected from 59 DRIFT2 participants (25 DCR and 34 IMF). 

### 3.2. Characteristics of the Study Participants and Clinical Health Measures from Baseline to Three Months

**Figure 2:**
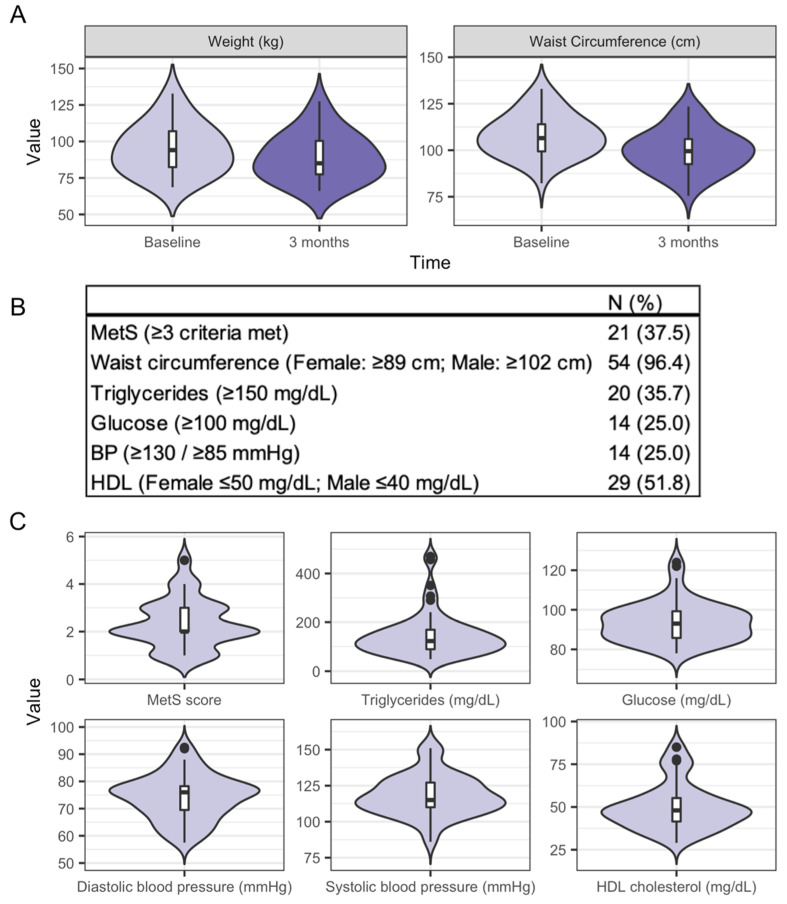
Clinical characteristics of DRIFT2 participants. (**A**) From baseline to three months of the intervention, participants (*n =* 59) lost significant weight and waist circumference as shown in these violin plots. (**B**) Blood-based clinical health measures for DRIFT2 participants were assessed at baseline but not at three months. This tables shows the number of participants meeting each of the criteria for metabolic syndrome (MetS) at baseline (*n* = 56). (**C**) These violin plots show the distribution of metabolic syndrome (MetS) score (*n* = 56), which is the total number of components that define MetS that exceed the thresholds shown in (**B**), and of the components of MetS. BP: blood pressure; MetS: metabolic syndrome; HDL: high density lipoprotein.

Table 1 shows the baseline characteristics of the participants in this ancillary study. The participants were predominantly female (76.3%), White (89.8%) with a mean age of 40.7 years (SD: 9.8), weight of 94.4 kg (SD: 16.0) and BMI of 33.1 kg/m^2^ (SD: 4.4), with no significant differences in baseline characteristics between the intervention groups. Anthropometric measures were assessed at baseline and three months, which coincides with the gut microbiota data used in this study. At three months, over half of the participants in this ancillary study (*n* = 31; 59.6%) had lost at least 5% of their baseline weight, and 13.4% (*n* = 7) had lost 10% of their baseline weight. On average, participants lost 5.8 ± 3.8 kg with a corresponding decrease in waist circumference of 8.3 ± 5.7 cm (Figure 2A). Clinical health measures at three months are not reported by the intervention group because the trial is ongoing and primary study investigators are not yet unblinded. Metabolic syndrome (MetS), as defined by the Adult Treatment Panel III (ATP-III) [44], was present at baseline in 37.5% of participants (Figure 2B). The distribution of MetS score, which is the number of components of MetS that exceed the thresholds shown in Figure 2B, and its components are shown in Figure 2C. There were no significant differences between the intervention groups in these clinical measures at baseline (Table S1).

### 3.3. Gut Microbiota of Participants Shifted Significantly in Composition during the First Three Months of the Intervention

**Figure 3:**
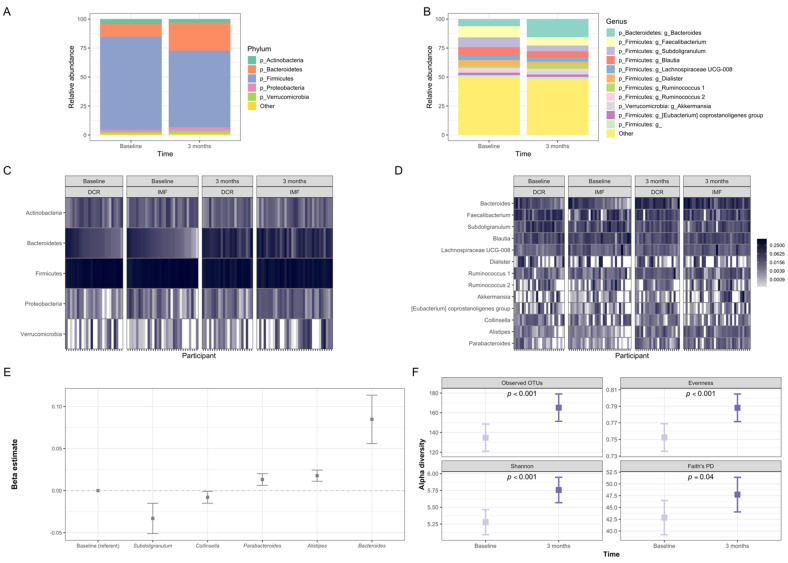
Changes in the overall gut microbiota of DRIFT2 participants from baseline to three months. The overall gut microbiota community structure (beta-diversity) shifted significantly from baseline to three months (*p* < 0.001 for weighted and unweighted UniFrac metrics). (**A**) Average relative abundance of the most prevalent gut microbiota phyla among study participants at baseline (*n =* 56) and three months (*n =* 55). (**B**) Average relative abundance among study participants at baseline (*n =* 56) and three months (*n =* 55) of the most prevalent gut microbiota genera and those that changed significantly from baseline to three months. (**C**) Relative abundance of the most prevalent gut microbiota phyla for DCR and IMF study participants at baseline (*n =* 25 DCR/31 IMF) and three months (*n =* 22 DCR / 33 IMF). (**D**) Relative abundance of the most prevalent gut microbiota genera and those that changed significantly for DCR and IMF study participants at baseline (*n =* 25 DCR / 31 IMF) and three months (*n =* 22 DCR/33 IMF). (**E**) Gut microbiota taxa showing significant (FDR *p* < 0.1) overall change from baseline to three months. The y-axis shows the regression estimate from longitudinal models (using the ANCOM method, controlling for age, sex, intervention group, time and an interaction between intervention group and time, for *n =* 104 samples from 52 individuals) for the scaled proportion relative abundance of each taxa (with one unit corresponding to one standard deviation) on the x-axis at three months relative to baseline. **(F)** These plots show the change from baseline to three months for four indices of alpha-diversity: Observed OTUs, Evenness, Shannon diversity index, and Faith’s Phylogenetic Diversity. These indices reflect different aspects of diversity, such as the richness, evenness, and phylogenetic relatedness of the organisms detected in the samples. All alpha-diversity indices increased significantly over the first three months of the intervention based on linear mixed models of the 111 samples from 59 individuals, controlling for age, sex, intervention group, and time (interactions between intervention group and time were not significant and, thus, not included). OTUs: operational taxonomic units (clusters of organisms that are grouped by gene sequence similarity); PD: Phylogenetic Diversity.

Gut microbiota of participants at baseline and three months was dominated by the phyla Firmicutes and Bacteroidetes (Figure 3A,B), as is commonly observed in Western populations [45]. The most common genera at both time points were *Faecalibacterium, Subdoligranulum, Blautia* and *Bacteroides* (Figure 3C,D). The overall microbiota community structure (beta-diversity) shifted significantly from baseline to three months, controlling for age, sex and intervention group assignment (*p* ≤ 0.001; Figure 3A–D). Using the analysis of composition of microbiomes (ANCOM) method [37], we observed five bacterial genera that changed in relative abundance from baseline to three months (*Subdoligranulum, Collinsella, Parabacteroides*, *Alistipes,* and *Bacteroides*; Figure 3E). *Subdoligranulum* and *Collinsella* decreased in relative abundance, while the other three taxa increased. The diversity of microbiota also increased from baseline to three months, as evidenced by increases in four alpha-diversity indices that captured different aspects of the richness and evenness of distribution of the types of microorganisms detected in each sample (Figure 3F).

### 3.4. The Overall Gut Microbiota Composition Was Significantly Associated with Clinical Health Measures in Cross-Sectional Analyses

**Figure 4:**
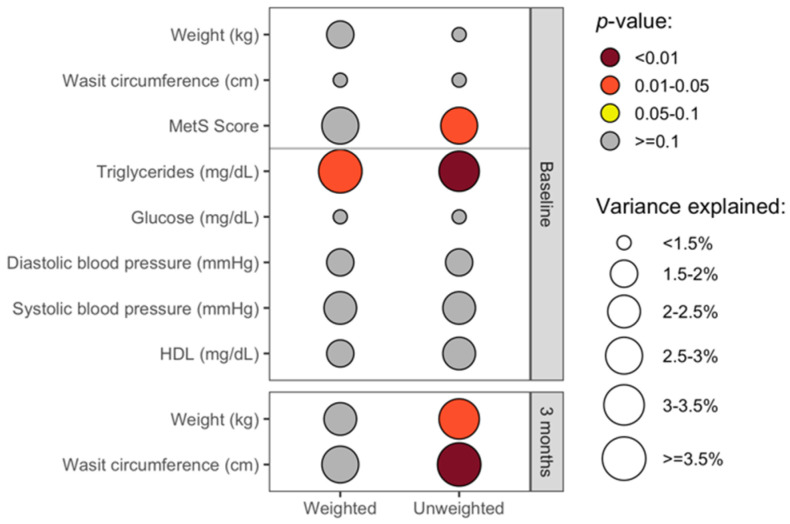
Cross-sectional associations between the gut microbiota and clinical health measures. This plot shows the cross-sectional associations between clinical health measures and the overall gut microbiota composition at baseline (upper panel; *n =* 56) and three months (lower panel; *n =* 55) based on permutational ANOVA models, controlling for age and sex, as well as intervention group for analyses of data from three months. The color shows the p-value for the association, and the size of the circles represents the amount of variation explained (R^2^) in the overall gut microbiota composition as quantified by weighted (left) and unweighted (right) UniFrac metrics. While numerous clinical measures were collected at baseline, including all of the components of MetS, weight and waist circumference were the primary clinical measures collected at three months.

The overall gut microbiota community structure at baseline, as quantified by weighted and unweighted UniFrac beta-diversity indices, did not significantly correlate with demographic factors, including age, sex, race, ethnicity, income, or marital status (R^2^ ≤ 2.1%; *p* > 0.36). Likewise, weight and waist circumference did not significantly correlate with the beta-diversity metric at baseline (Figure 4). The MetS score was associated at baseline with the unweighted UniFrac beta-diversity metric of the overall gut microbiota community structure. Unweighted UniFrac is a qualitative measure of the gut microbiota composition, reflecting the types of microbes present, whereas weighted UniFrac reflects the types and abundance of microbes [46]. We explored the association with the MetS score further by examining each of its components, and we observed that baseline triglyceride levels were associated with both weighted and unweighted beta-diversity metrics. At three months, the blood-based clinical components of MetS were not measured, but both weight and waist circumference were significantly associated with unweighted UniFrac, but not with weighted (Figure 4). 

### 3.5. Baseline Gut Microbiota Composition Predicts Change in Waist Circumference at 3 Months

In order to evaluate the potentially contributory role of the gut microbiota towards weight loss, we examined the relationship between the baseline gut microbiota beta-diversity and percent change in body weight and in waist circumference from baseline to three months. The beta-diversity metrics did not show significant associations with percent change in weight, but the weighted UniFrac metric was significantly predictive of percent change in waist circumference (*p* = 0.01; Table S2). We also examined these associations with the change in gut microbiota community structure from baseline to three months based on UniFrac metrics, and the results were similar, with no detectible association with weight change but a strong association between the weighted UniFrac metric and change in waist circumference (*p* = 0.009; Table S2). We did not observe significant associations between alpha-diversity and these outcomes (Table S3). 

### 3.6. Baseline and Changes in Specific Gut Microbiota Genera Were Associated with Change in Weight and Waist Circumference at 3 Months

**Figure 5:**
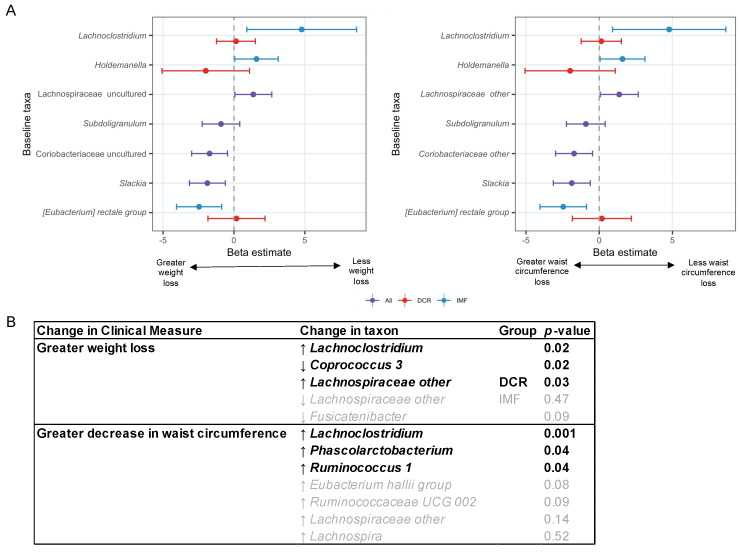
Associations between the gut microbiota and change in clinical health measures. (**A**) Abundance of the plotted genera were identified as the most predictive at baseline of percent change in weight and in waist circumference from baseline to three months using a random forest-based prediction method called VSURF (*n =* 56). Linear regression modeling (controlling for age, sex, and intervention group, and evaluating the interaction between the taxon and intervention group) was used to help interpret the results. The forest plot shows regression estimates (x-axis) for one standard deviation change in the relative abundance of the taxon in models predicting percent change in the clinical measure. Lower beta values indicate that greater abundance of the taxon correlate with greater decreases in the clinical measure and vice versa. Some of the selected taxa show different relationships with the outcomes among DCR (red) versus IMF (blue). For example, greater abundance of *Subdoligranulum* was associated with greater weight loss among IMF but not among DCR. Some selected predictors do not show statistically significant linear relationships with change in the clinical measure, but they may interact with each other in complex ways in relation to the outcome. “Other” genera indicate sequences that could only be classified to the family-level. (**B**) Change in the abundance of the shown taxa from baseline to three months were identified as the most predictive of percent change in weight and in waist circumference from baseline to three months using a random forest-based prediction method called VSURF (*n =* 52). Linear regression modeling (controlling for age, sex, and intervention group, and evaluating the interaction between the taxon and intervention group) was used to help interpret the results. *Lachnospiraceae other* showed a different relationship with percent weight loss among DCR versus IMF, with increases in abundance associated with greater weight loss only among DCR. While some selected predictors do not show statistically significant linear relationships with change in the clinical measure (shown in grey), these selected taxa may interact with each other in complex ways in relation to the outcome. “Other” genera indicate sequences that could only be classified to the family-level. MetS: Metabolic syndrome; HDL: High density lipoprotein; DCR: Daily caloric restriction; IMF: Intermittent fasting.

We used random forests with feature selection techniques [42] to identify whether specific bacterial genera at baseline were predictive of percent change in body weight and in waist circumference at three months. Model metrics are presented in Table S4. Four genera at baseline were selected as being jointly predictive of percent weight change. Random forests allow for modeling of complex relationships among the selected predictors and between predictors and the outcome. Hence, we do not expect all of these identified predictors to individually show direct linear relationships with the outcomes, but we used linear regressions in order to assist with interpretation of the findings. In the regression models, we controlled for age, sex and randomized group assignment, and we assessed whether there was a significant interaction between the predictor and intervention group. The results of these regression models are shown in Figure 5A. Most of the identified genera did not show significant linear relationships with weight loss; one exception was that greater abundance of *Subdoligranulum* was associated with greater weight loss among IMF but not DCR. Seven genera at baseline were identified in the random forests as predictive of percent change in waist circumference (Table S4; Figure 5A). In regression models of the identified taxa, greater abundance of *Coriobacteriaceae other* and *Slackia* at baseline were associated with larger decreases in waist circumference; greater baseline abundance of *Lachnospiraceae other* was associated with less decrease in waist circumference. Greater baseline abundance of *[Eubacterium] rectale group* was associated with greater waist circumference loss among IMF, whereas greater baseline abundance of *Holdemanella* and *Lachnoclostridium* were associated with less waist circumference loss among IMF. 

We applied a similar approach of random forests followed by regression models to the *change* in genera from baseline to three months (Table S4). Changes in the abundance of four genera from baseline to three months were identified as associated with percent weight change (Figure 5B). An increase in the abundance of *Lachnoclostridium* and a decrease in *Coprococcus 3* and *Fusicatenibacter* were associated with greater weight loss; increased *Lachnospiraceae other* abundance was associated with greater weight loss in DCR but not in IMF. Changes in the abundance of seven genera were identified as associated with percent waist circumference change (Table S4; Figure 5B). Increase in the abundance of *Lachnoclostridium, Phascolarctobacterium*, and *Ruminococcus 1* was associated with greater waist circumference loss. 

### 3.7. In Subset Analyses, Change in Energy Intake Was Predictive of Change in Gut Microbiota Beta-Diversity 

The relationship between the gut microbiota and weight loss is complex. The underlying assumption of most of the preceding analyses of clinical outcomes is that the gut microbiota impacts these measures—possibly through direct effects or through a mediating role between the intervention (involving changes in diet and physical activity) and changes in these outcomes. However, it is also possible that weight loss causes the gut microbiota to change (reverse causality). Thus, it is important to assess the potential impacts of changes in weight-related health behaviors targeted by the behavioral weight loss intervention (i.e., dietary intake and physical activity) on the gut microbiota, and to examine if the relationships seen above between the gut microbiota and clinical outcomes reported above are independent of diet and physical activity. 

In order to evaluate the change in dietary intake and physical activity as predictors of change in the gut microbiota, we used permutational ANOVA of the change in beta-diversity (based on UniFrac metrics) from baseline to three months among the *n* = 45 individuals with complete microbiota and dietary information at baseline and three months, and the *n* = 42 individuals who also had valid physical activity data at these timepoints. We examined the change in average energy intake (kcal/day) and in percent intake of macronutrients based on seven-day diet records. We also examined the change in time (min/day) spent engaging in moderate to vigorous physical activity (defined as stepping cadence ≥ 75 steps/minute) [29] and sitting time (min/day) based data from the activPAL v4 device (PAL Technologies, Glasgow, Scotland) worn for 1 week (Figure S1A). The results are shown in Figure S1B,C. We found that in models including change in weight and in diet (controlling for age, sex and intervention group), the change in energy intake was significantly predictive of change in the gut microbiota beta-diversity, as measured by unweighted UniFrac; in contrast, weight change was not significant. The results were similar for change in the waist circumference. In beta-diversity models using weighted UniFrac and in models of change in physical activity, none of the included predictors were statistically significant. Although alpha-diversity changed significantly over the time period evaluated (Figure 3E), none of the examined predictors (change in diet or physical activity) were significantly associated with the changes in alpha-diversity indices (Table S5).

Given the evidence of association between energy restriction and the gut microbiota as well as between the gut microbiota and change in weight and waist circumference, we next assessed the association between change in dietary energy intake and change in weight and waist circumference since the gut microbiota may mediate links between these factors (focusing on energy intake as this was the only dietary variable showing an association with overall GM composition); however, no evidence for an association between these variables was noted (*p* ≥ 0.14). When additionally examining macronutrients, only protein showed an association with increased protein associated with greater loss in waist circumference (*p* = 0.044). 

In the analyses described above of gut microbiota beta-diversity metrics as predictors of clinical outcomes, we controlled for these same dietary and physical activity measures in the subsets of individuals where this information was available. The relationships described above persisted (Table S6).

### 3.8. Analyses Comparing DCR to IMF Reveal Differences in the Gut Microbiota over Time and the Relationship with Clinical Measures

**Figure 6:**
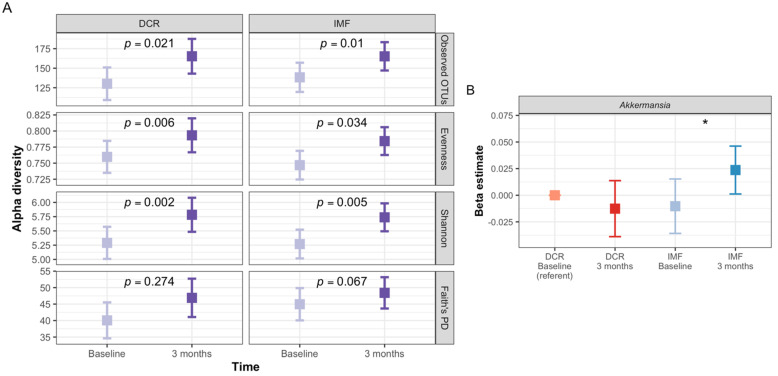
Changes in the gut microbiota of DRIFT2 participants from baseline to three months by intervention group. (**A**) This plots shows the change from baseline to three months by intervention group of four indices of alpha-diversity: Observed OTUs, Evenness, Shannon diversity index, and Faith’s Phylogenetic Diversity (PD), which reflect different aspects of alpha-diversity, such as the richness, evenness, and phylogenetic relatedness of the organisms detected in the samples. All alpha-diversity indices, except Faith’s PD, showed significant increases over time in both DCR (*n =* 47 samples from 25 individuals) and IMF (*n =* 64 samples from 34 individuals) groups based on linear mixed models by intervention group, controlling for age, sex, and time. There were not significant differences between intervention groups (DCR versus IMF) in the change in alpha-diversity over time (interaction *p*-values ≥ 0.48). (**B**) Only one gut microbiota taxon (*Akkermansia*) showed significant differences between intervention groups in terms of the relative abundance change from baseline to three months. The y-axis shows the regression estimate from longitudinal models (using the ANCOM method using the ANCOM method, controlling for age, sex, intervention group, time, and an interaction between intervention group and time, for *n =* 104 samples from 52 individuals; 22 DCR and 30 IMF) for the change in proportion relative abundance relative to DCR at baseline. The * indicates significant change over time within the intervention group.

Since DRIFT2 is an ongoing randomized trial, we cannot report the clinical outcomes by randomized intervention group due to the blinding of study investigators to intervention group-specific outcomes. Understanding the between-group differences in the clinical outcomes is part of the interpretation of between-group differences in the gut microbiota over time. Thus, we focused our primary analyses on overall changes in the gut microbiota, while controlling for the intervention group in the longitudinal analyses and assessing time-by-group interactions. The change in the overall beta- and alpha-diversity metrics did not show significant group by time interactions (Table 2, Figure 6A and Figure S2). One taxon, *Akkermansia*, showed significant between-group differences in changes from baseline to three months (Figure 6B), increasing significantly among IMF participants but showing no significant change among DCR. 

We examined whether the association between beta-diversity of the gut microbiota at baseline and percent change in waist circumference observed in the overall cohort was evident in each intervention group. The associations between the baseline beta-diversity and waist circumference may have been driven predominantly by IMF participants (Table S2). 

## 4. Discussion

In this ancillary study, we examined the gut microbiome and its relationship to clinical outcome measures in individuals who are overweight or obese, undergoing a lifestyle weight loss intervention, focusing on either DCR or IMF as the dietary energy-restriction strategy. We focused on the first three months of intervention when dietary adherence was likely highest [21]. During this time, most participants lost a clinically significantly amount of weight (~60% lost at least 5% of initial body weight). Our results provide evidence that there are significant early changes in gut microbiota profiles in response to dietary energy restriction, that some of these changes may differ between DCR and IMF, and that the gut microbiota composition correlates with clinical health metrics and may contribute towards responsiveness to the interventions. Future research in this cohort will help to clarify the persistence of these microbial changes and the durability of these associations, as well as the relationship between these changes and clinical outcomes within the DCR and IMF groups. 

In this study, we observed significant changes from baseline to three months in the types and abundances of microbes present in the gut among both DCR and IMF intervention groups. We also observed changes in specific genera, including increases in *Bacteroides* and *Alistipes*, and decreases in *Collinsella*. Increased abundance of *Bacteroides* has been previously noted with hypocaloric weight-loss diets [47,48,49], although this association has not been consistently observed [17]. Increases in the *Alistipes* genus have been observed following surgical weight loss interventions [17], and higher baseline *Alistipes* abundance has been correlated with greater success in long-term weight-loss maintenance following a dietetic and lifestyle intervention [50]. Reductions in *Collinsella* abundance have been noted during a structured hypocaloric weight loss program in a small cohort of type 2 diabetics with obesity [51], as well in a reduced carbohydrate weight loss intervention of men who were overweight [52].

In this study, *Subdoligranulum* also decreased during the intervention. This genus has only one species, *S. variabile,* which has been previously associated with metabolic syndrome. Interestingly, in an intervention study of fecal microbiota transplantation from lean donors to men with metabolic syndrome [53], higher baseline *S. variabile* abundance was predictive of greater improvements in insulin sensitivity. We likewise observed that baseline *Subdoligranulum* was among the genera most predictive of change in weight and waist circumference and that higher baseline abundance of *Subdoligranulum* was predictive of greater weight loss among IMF participants (though not DCR). However, a direct linear relationship was not found between baseline *Subdoligranulum* and loss in waist circumference, suggesting that this genus is likely important in combination with other taxa. Baseline abundances of two members of the family Coriobacteriaceae (*Slackia* and an unclassified genus) were also predictive at baseline of greater loss in waist circumference. This family has been linked to multiple adverse phenotypes, including obesity [54]; however, higher baseline levels were also identified as contributing towards the beneficial effects of Roux-en-Y gastric bypass among people with type 2 diabetes [55]. Furthermore, *Slackia* may help to increase the bioavailability of polyphenols, which would be beneficial for cardiometabolic health [56]. 

We also examined whether changes in specific microbial taxa were predictive of change in weight and waist circumference since the intervention could cause beneficial changes in gut microbiota taxa that then contribute towards improvements in these clinical outcomes. Changes in the abundances of numerous genera were associated with enhanced loss of weight or waist circumference. For instance, a decrease in the abundance of the genus *Coprococcus 3* was associated with greater weight loss. Prior work has shown that this genus was enriched among Mexican women with obesity and with metabolic syndrome, compared to normal weight controls [57] and among Chinese youth and Japanese adults with obesity [58,59]. Decreases in abundance of this genus have also been noted with Laparoscopic sleeve gastrectomy [60] and a weight loss intervention among adolescents [48]. In our study, an increase in the genus *Phascolarctobacterium* was associated with greater decreases in waist circumference. This is consistent with a prior study that found a negative correlation between *Phascolarctobacterium* and percent body fat [61], which the authors thought could reflect the relationship between excess body fat and decreased insulin sensitivity since *Phascolarctobacterium* is likewise correlated with higher insulin sensitivity. However, other studies have shown inconsistent relationships between *Phascolarctobacterium* and markers of insulin sensitivity (as discussed in Naderpoor et al., 2019). In this study, as well as in a prior multi-omic study using this same cohort, higher *Lachnospiraceae other* abundance at baseline was associated with less reduction in waist circumference [23], yet we also observed that an increase from baseline to three months in this taxon was actually associated with greater weight loss among DCR participants. This is a heterogeneous group with potentially diverse functional effects, and these associations should be further explored, ideally using shotgun metagenomic sequencing data to resolve species/strain-level taxonomy.

Increases in alpha-diversity have previously been noted during hypocaloric weight loss interventions [20,51], as observed in our study. Lower microbial diversity has been associated with numerous cardiometabolic health conditions and risk factors, including diabetes [62], non-alcoholic fatty liver disease [63], and obesity [64]. Prior research suggests that decreased diversity may only be associated with obesity in a subset of individuals [65,66]. Interestingly, individuals with low baseline alpha-diversity may show greater responsiveness to interventions aiming to improve cardiometabolic health, as was seen in interventions involving fecal microbiota transfer from lean donors to individuals with obesity [53,67]. We did not observe significant associations between changes in alpha-diversity and change in weight or waist circumference, although future work in a larger sample could specifically examine these relationships among individuals with low baseline alpha-diversity. We were unable to identify statistically significant predictors of the change in alpha-diversity, such as dietary macronutrient composition or summary measures of exercise. All participants in the trial received counseling in healthy food choices and a prescription to gradually increase physical activity. It is possible that the observed increases in alpha-diversity were due to a combination of these lifestyle changes that are not apparent when examining these factors individually or when using broad summary metrics of diet and exercise. Again, a larger sample size may improve our ability to understand these relationships.

This is the first study to examine gut microbiota during a lifestyle weight loss intervention of DCR versus IMF as well as the largest human study to date of the gut microbiota and IMF. Studies of IMF have documented that there may be enhanced health benefits relative to DCR for many measures related to cardiometabolic health, including body composition, resting energy expenditure, insulin sensitivity and inflammation [68,69,70,71,72]. We examined whether changes in gut microbiota differed between the two intervention arms, and we did not see significant differences between DCR and IMF in terms of how the overall gut microbiota changed over time (alpha- and beta-diversity). The genus *Akkermansia* increased significantly with IMF but did not change with DCR. While this difference could reflect differences in weight loss or other clinical health metrics, it could also be due to differential effects of more extended time periods of fasting with IMF. *Akkermansia* only contains two known species, the most common of which, *A. muciniphila*, is a mucin-degrading bacterium that has been causally linked in animal models to lowering body fat mass, improving glucose homoeostasis, decreasing adipose tissue inflammation and increasing gut integrity, as well as cardiometabolic improvements during dietary energy restriction [73,74,75]. *Akkermansia* is also an important producer of acetate, a short-chain fatty acid that may be a key mediator of gut microbiota-dependent changes in the composition of adipose tissue, specifically “beiging” of white adipose tissue, previously reported in animal models of IMF [19]. Beiging is a process in which white adipose tissue takes on a more brown-like phenotype, and it has important beneficial effects on insulin sensitivity and resting energy expenditure [76]. Oral administration of *A. muciniphila* has likewise been shown to promote beiging [77]. 

The gut microbiome is involved in extensive crosstalk with the host metabolic and immune systems [78]. While some evidence supports the causal role of the gut microbiota in obesity [7,8,9,10,11], gut microbiota also respond to changes in the environment (nutritional, chemical and behavioral) [78]. Thus, gut microbiota may contribute towards weight loss but may also shift in response to weight loss, and it is difficult to establish with confidence the predominant direction of this relationship [79]. In this study, we assessed predictors of the observed change in the gut microbiota composition during the intervention and found that change in energy intake was the most significant driver of changes in the types of microbes present in the gut (beta-diversity based on unweighted UniFrac), yet weight loss was not a significant predictor of changes in the overall gut microbiota composition. While these results are consistent with the hypothesis that dietary changes impact gut composition and the changes in the gut microbiome then contribute to weight loss, we did not observe evidence to support the second part of this mediation pathway, i.e., we did not observe an association between the overall gut microbiota composition (beta-diversity) and weight loss. We did, however, observe that baseline abundance of subsets of genera were predictive of weight loss, and likewise that shifts in some genera were associated with weight loss, as discussed above. There is growing evidence to support the role of gut microbiota as a mediator of the effects of diet on the host metabolic status [80], and specifically of the effects of dietary energy restriction on metabolic improvements [19,81]. We also examined the association between change in physical activity and shifts in the gut microbiota composition. Although some prior studies support that increases in aerobic physical activity (both with and without resistance training) impact gut composition [82], we did not see this association.

This study has both strengths and limitations. The parent trial is a rigorously designed interventional weight loss trial, comparing IMF to DCR, and the dietary interventions are designed to produce an equivalent weekly dietary energy deficit. This ancillary study is the only study to date to examine gut microbial diversity and composition during a behavioral weight loss trial comparing weight loss generated by IMF to DCR. Use of short-read 16S rRNA amplicon sequences to profile gut microbiota limited our ability to examine gut microbiota in terms of either species/strain-level shifts or changes in functional capacity. This work is based on the first three months of data from two of five recruitment cohorts from the parent trial; thus, the sample size is relatively small. Since the parent trial is ongoing, we are not able to report differences in the clinical outcomes between the DCR and IMF intervention groups, which limits our ability to interpret any differences in the gut microbiome of these groups. The timing of food intake during IMF may impact the clinical improvements associated with this approach [83], and the timing of the stool collection (in a fasted versus fed state/time of day) and recent antibiotic use may impact the gut microbiota [19,84], but this information was missing for many of the samples. Self-reported dietary intake also suffers from considerable inaccuracies and bias [85]. Despite these limitations and possible heterogeneity in the data, our findings show significant and clinically relevant associations that will inform future work, including a more thorough comparison of changes in gut microbiota features with DCR versus IMF once the trial is unblinded.

## 5. Conclusions

This work adds to the growing body of literature demonstrating that the gut microbiota plays an important role in body weight regulation and may contribute towards responsiveness during a weight loss intervention. During the first three months of a lifestyle weight loss intervention involving an energy-restricted diet and increased physical activity, gut microbiota of participants changed significantly. Of particular note, we found that the baseline gut microbiota composition was predictive of change in waist circumference at three months and that numerous bacterial taxa were associated with improvements in weight and waist circumference measures. This suggests that the gut microbiota community structure may influence responsiveness to weight loss efforts, which is critical to understand more fully, as gut microbiota profiles are alterable through various means, such as probiotics/prebiotics [13], personalized dietary changes [86] or targeting gut microbiota pathways and metabolites [87]. This is an exciting avenue for further research.

## Figures and Tables

**Table 1:** Characteristics of study cohort by intervention group. There were no significant differences in characteristics between the intervention groups at baseline.

	Overall	DCR	IMF	*p*-Value
*n*	59	25	34	
Age (mean (SD))	40.7 (9.8)	42.0 (10.4)	39.8 (9.3)	0.384
Female sex	45 (76.3)	18 (72.0)	27 (79.4)	0.725
Race				>0.99
White	53 (89.8)	22 (88.0)	31 (91.2)	
Black or African American	4 (6.8)	2 (8.0)	2 (5.9)	
Asian	2 (3.4)	1 (4.0)	1 (2.9)	
Hispanic ethnicity	10 (16.9)	6 (24.0)	4 (11.8)	0.297
Education				0.642
1 year College	1 (1.7)	1 (4.0)	0 (0.0)	
2 year College	2 (3.4)	0 (0.0)	2 (5.9)	
3 year College	3 (5.1)	1 (4.0)	2 (5.9)	
4 year College	25 (42.4)	9 (36.0)	16 (47.1)	
Master’s Degree	22 (37.3)	11 (44.0)	11 (32.4)	
Doctorate Degree	6 (10.2)	3 (12.0)	3 (8.8)	
Income				0.237
<25,000	5 (8.5)	2 (8.0)	3 (8.8)	
25,000–40,000	5 (8.5)	1 (4.0)	4 (11.8)	
45,000–70,000	12 (20.3)	5 (20.0)	7 (20.6)	
70,000–110,000	15 (25.4)	10 (40.0)	5 (14.7)	
>110,000	22 (37.3)	7 (28.0)	15 (44.1)	
Marital status				0.113
Single	10 (16.9)	2 (8.0)	8 (23.5)	
Committed Relationship	8 (13.6)	2 (8.0)	6 (17.6)	
Married	35 (59.3)	17 (68.0)	18 (52.9)	
Divorced	5 (8.5)	4 (16.0)	1 (2.9)	
Widowed	1 (1.7)	0 (0.0)	1 (2.9)	
Body mass index (kg/m^2^)	33.1 (4.4)	32.9 (4.7)	33.2 (4.1)	0.803
Stool collection				
Stool at baseline	56 (94.9)	25 (100.0)	31 (91.2)	0.355
Stool at 3 months	55 (93.2)	22 (88.0)	33 (97.1)	0.399
Stool at both times	52 (88.1)	22 (88.0)	30 (88.2)	>0.99

**Table 2:** Results of longitudinal permutational ANOVA models to assess whether changes in the overall gut microbiota composition over the first three months of the intervention differed by group (DCR versus IMF). Quantitative (weighted UniFrac) and qualitative (unweighted UniFrac) indices of beta-diversity were used as summary measures of the gut microbiota composition among *n =* 111 samples from 59 individuals (47 samples from 25 DCR / 64 samples from 34 IMF). There were no significant differences between intervention groups in the change in beta-diversity over time.

Metric	Covariate	F	*p*-Value
Weighted UniFrac	Time	8.3	<0.001
	Group	1.7	0.970
	Group*Time	0.6	0.474
Unweighted UniFrac	Time	1.7	0.001
	Group	2.0	0.650
	Group*Time	0.8	0.118

## Data Availability

The data used in this study are available from the European Bioinformatics Institute (EBI), accession No. PRJEB44113 (ERP128124).

## Supplementary Materials

The following are available online at https://www.mdpi.com/article/10.3390/nu13093248/s1, Figure S1. Change in diet and physical activity of DRIFT2 participants from baseline to three months and the association with change in the gut microbiota community structure (beta-diversity); Figure S2. Changes in the gut microbiota taxa of DRIFT2 participants from baseline to three months by intervention group assignment; Table S1. Clinical characteristics of study cohort by intervention group; Table S2. Association between baseline and change in participant gut microbiota composition and two clinical outcome measures, percent change in weight and in waist circumference; Table S3. Association between participant gut microbiota alpha-diversity and outcome measures; Table S4. Performance metrics of random forests to predict percent change in weight and in waist circumference using all taxa and those selected as most highly predictive of these outcomes by machine learning techniques; Table S5. Regression models of the change in alpha-diversity as a function of change in weight or waist circumference, change in dietary intake and change in physical activity; Table S6. Association between baseline and change in participant gut microbiota composition and two clinical outcome measures, percent change in weight and in waist circumference, controlling for change in dietary intake and physical activity.

## References

[1] Virani Salim S., Alvaro A., Benjamin Emelia J., Bittencourt Marcio S., Callaway Clifton W., Carson April P., Chamberlain Alanna M., Chang Alexander R., Cheng S., Delling Francesca N. (2020). Heart Disease and Stroke Statistics—2020 Update: A Report From the American Heart Association. Circulation.

[2] Mattson M.P., Wan R. (2005). Beneficial Effects of Intermittent Fasting and Caloric Restriction on the Cardiovascular and Cerebrovascular Systems. J. Nutr. Biochem..

[3] Jensen M.D., Ryan D.H., Apovian C.M., Ard J.D., Comuzzie A.G., Donato K.A., Hu F.B., Hubbard V.S., Jakicic J.M., Kushner R.F. (2014). 2013 AHA/ACC/TOS Guideline for the Management of Overweight and Obesity in Adults: A Report of the American College of Cardiology/American Heart Association Task Force on Practice Guidelines and The Obesity Society. J. Am. Coll. Cardiol..

[4] Marek R.J., Coulon S.M., Brown J.D., Lydecker J.A., Marek S., Malcolm R., O’Neil P.M. (2017). Characteristics of Weight Loss Trajectories in a Comprehensive Lifestyle Intervention. Obesity.

[5] Greaves C., Poltawski L., Garside R., Briscoe S. (2017). Understanding the Challenge of Weight Loss Maintenance: A Systematic Review and Synthesis of Qualitative Research on Weight Loss Maintenance. Health Psychol. Rev..

[6] Chen R., Snyder M. (2013). Promise of Personalized Omics to Precision Medicine. Wires Syst. Biol. Med..

[7] DiBaise J.K., Frank D.N., Mathur R. (2012). Impact of the Gut Microbiota on the Development of Obesity: Current Concepts. Am. J. Gastroenterol. Suppl..

[8] Maruvada P., Leone V., Kaplan L.M., Chang E.B. (2017). The Human Microbiome and Obesity: Moving beyond Associations. Cell Host Microbe.

[9] Sanna S., van Zuydam N.R., Mahajan A., Kurilshikov A., Vila A.V., Võsa U., Mujagic Z., Masclee A.A.M., Jonkers D.M.A.E., Oosting M. (2019). Causal Relationships among the Gut Microbiome, Short-Chain Fatty Acids and Metabolic Diseases. Nat. Genet..

[10] Torkamani A., Topol E. (2019). Polygenic Risk Scores Expand to Obesity. Cell.

[11] Turnbaugh P.J., Hamady M., Yatsunenko T., Cantarel B.L., Duncan A., Ley R.E., Sogin M.L., Jones W.J., Roe B.A., Affourtit J.P. (2009). A Core Gut Microbiome in Obese and Lean Twins. Nature.

[12] Klimentidis Y.C., Bea J.W., Lohman T., Hsieh P.-S., Going S., Chen Z. (2015). High Genetic Risk Individuals Benefit Less from Resistance Exercise Intervention. Int. J. Obes..

[13] Kunnackal G.J., Wang L., Nanavati J., Twose C., Singh R., Mullin G. (2018). Dietary Alteration of the Gut Microbiome and Its Impact on Weight and Fat Mass: A Systematic Review and Meta-Analysis. Genes.

[14] Turnbaugh P.J., Ley R.E., Mahowald M.A., Magrini V., Mardis E.R., Gordon J.I. (2006). An Obesity-Associated Gut Microbiome with Increased Capacity for Energy Harvest. Nature.

[15] Ridaura V.K., Faith J.J., Rey F.E., Cheng J., Duncan A.E., Kau A.L., Griffin N.W., Lombard V., Henrissat B., Bain J.R. (2013). Gut Microbiota from Twins Discordant for Obesity Modulate Metabolism in Mice. Science.

[16] Meijnikman A.S., Gerdes V.E., Nieuwdorp M., Herrema H. (2018). Evaluating Causality of Gut Microbiota in Obesity and Diabetes in Humans. Endocr. Rev..

[17] Seganfredo F.B., Blume C.A., Moehlecke M., Giongo A., Casagrande D.S., Spolidoro J.V.N., Padoin A.V., Schaan B.D., Mottin C.C. (2017). Weight-Loss Interventions and Gut Microbiota Changes in Overweight and Obese Patients: A Systematic Review: Weight-Loss Impact on Gut Microbiota. Obes. Rev..

[18] Wang S., Huang M., You X., Zhao J., Chen L., Wang L., Luo Y., Chen Y. (2018). Gut Microbiota Mediates the Anti-Obesity Effect of Calorie Restriction in Mice. Sci. Rep..

[19] Li G., Xie C., Lu S., Nichols R.G., Tian Y., Li L., Patel D., Ma Y., Brocker C.N., Yan T. (2017). Intermittent Fasting Promotes White Adipose Browning and Decreases Obesity by Shaping the Gut Microbiota. Cell Metab..

[20] Cotillard A., Kennedy S.P., Kong L.C., Prifti E., Pons N., Le Chatelier E., Almeida M., Quinquis B., Levenez F., Galleron N. (2013). Dietary Intervention Impact on Gut Microbial Gene Richness. Nature.

[21] Dansinger M.L., Gleason J.A., Griffith J.L., Selker H.P., Schaefer E.J. (2005). Comparison of the Atkins, Ornish, Weight Watchers, and Zone Diets for Weight Loss and Heart Disease Risk Reduction—A Randomized Trial. JAMA.

[22] Ross R., Neeland I.J., Yamashita S., Shai I., Seidell J., Magni P., Santos R.D., Arsenault B., Cuevas A., Hu F.B. (2020). Waist Circumference as a Vital Sign in Clinical Practice: A Consensus Statement from the IAS and ICCR Working Group on Visceral Obesity. Nat. Rev. Endocrinol..

[23] Siebert J.C., Stanislawski M.A., Zaman A., Ostendorf D.M., Konigsberg I.R., Jambal P., Ir D., Bing K., Wayland L., Scorsone J.J. (2021). Multiomic Predictors of Short-Term Weight Loss and Clinical Outcomes During a Behavioral-Based Weight Loss Intervention. Obesity.

[24] Donnelly J.E., Blair S.N., Jakicic J.M., Manore M.M., Rankin J.W., Smith B.K. (2009). Appropriate Physical Activity Intervention Strategies for Weight Loss and Prevention of Weight Regain for Adults. Med. Sci. Sports Exerc..

[25] Leavitt M.O. (2008). 2008 Physical Activity Guidelines for Americans.

[26] Peters J.C., Wyatt H.R., Foster G.D., Pan Z., Wojtanowski A.C., Veur S.S.V., Herring S.J., Brill C., Hill J.O. (2014). The Effects of Water and Non-Nutritive Sweetened Beverages on Weight Loss during a 12-Week Weight Loss Treatment Program. Obesity.

[27] Wyatt H.R., Jortberg B.T., Babbel C., Garner S., Dong F., Grunwald G.K., Hill J.O. (2008). Weight Loss in a Community Initiative That Promotes Decreased Energy Intake and Increased Physical Activity and Dairy Consumption: Calcium Weighs-In. J. Phys. Act. Health.

[28] Atkin A.J., Gorely T., Clemes S.A., Yates T., Edwardson C., Brage S., Salmon J., Marshall S.J., Biddle S.J. (2012). Methods of Measurement in Epidemiology: Sedentary Behaviour. Int. J. Epidemiol..

[29] Lyden K., Keadle S.K., Staudenmayer J., Freedson P.S. (2017). The ActivPALTM Accurately Classifies Activity Intensity Categories in Healthy Adults. Med. Sci. Sports Exerc..

[30] Jobira B., Frank D.N., Pyle L., Silveira L.J., Kelsey M.M., Garcia-Reyes Y., Robertson C.E., Ir D., Nadeau K.J., Cree-Green M. (2020). Obese Adolescents with PCOS Have Altered Biodiversity and Relative Abundance in Gastrointestinal Microbiota. J. Clin. Endocrinol. Metab..

[31] Soderborg T.K., Clark S.E., Mulligan C.E., Janssen R.C., Babcock L., Ir D., Young B., Krebs N., Lemas D.J., Johnson L.K. (2018). The Gut Microbiota in Infants of Obese Mothers Increases Inflammation and Susceptibility to NAFLD. Nat. Commun..

[32] Bolyen E., Rideout J.R., Dillon M.R., Bokulich N.A., Abnet C.C., Al-Ghalith G.A., Alexander H., Alm E.J., Arumugam M., Asnicar F. (2019). Reproducible, Interactive, Scalable and Extensible Microbiome Data Science Using QIIME 2. Nat. Biotechnol..

[33] Quast C., Pruesse E., Yilmaz P., Gerken J., Schweer T., Yarza P., Peplies J., Glöckner F.O. (2013). The SILVA Ribosomal RNA Gene Database Project: Improved Data Processing and Web-Based Tools. Nucleic Acids Res..

[34] Janssen S., McDonald D., Gonzalez A., Navas-Molina J.A., Jiang L., Xu Z.Z., Winker K., Kado D.M., Orwoll E., Manary M. (2018). Phylogenetic Placement of Exact Amplicon Sequences Improves Associations with Clinical Information. mSystems.

[35] Lozupone C., Lladser M.E., Knights D., Stombaugh J., Knight R. (2011). UniFrac: An Effective Distance Metric for Microbial Community Comparison. ISME J..

[36] Hu Y.-J., Satten G.A. (2020). Testing Hypotheses about the Microbiome Using the Linear Decomposition Model (LDM). Bioinformatics.

[37] Mandal S., Van Treuren W., White R.A., Eggesbø M., Knight R., Peddada S.D. (2015). Analysis of Composition of Microbiomes: A Novel Method for Studying Microbial Composition. Microb. Ecol. Health Dis..

[38] Benjamini Y., Hochberg Y.J.R. (1995). Controlling the False Discovery Rate: A Practical and Powerful Approach to Multiple Testing. J. R. Stat. Soc. Ser. B.

[39] Oksanen J., Blanchet F.G., Friendly M., Kindt R., Legendre P., McGlinn D., Minchin P.R., O’Hara R.B., Simpson G.L., Solymos P. (2018). Vegan: Community Ecology Package. https://CRAN.R-project.org/package=vegan.

[40] Zhao N., Chen J., Carroll I.M., Ringel-Kulka T., Epstein M.P., Zhou H., Zhou J.J., Ringel Y., Li H., Wu M.C. (2015). Testing in Microbiome-Profiling Studies with MiRKAT, the Microbiome Regression-Based Kernel Association Test. Am. J. Hum. Genet..

[41] Plantinga A.M., Chen J., Jenq R.R., Wu M.C. (2019). Pldist: Ecological Dissimilarities for Paired and Longitudinal Microbiome Association Analysis. Bioinformatics.

[42] Genuer R., Poggi J.-M., Tuleau-Malot C. (2015). VSURF: An R Package for Variable Selection Using Random Forests. R J..

[43] Williams C.K., Engelhardt A., Cooper T., Mayer Z., Ziem A., Scrucca L., Tang Y., Candan C., Kuhn M.M. (2016). Package ‘Caret’. https://CRAN.R-project.org/package=caret.

[44] Grundy M.S., Cleeman I.J., Daniels R.S., Donato A.K., Eckel H.R., Franklin A.B., Gordon J.D., Krauss M.R., Savage J.P., Smith C.S. (2005). Diagnosis and Management of the Metabolic Syndrome. Circulation.

[45] Human Microbiome Project Consortium (2012). Structure, Function and Diversity of the Healthy Human Microbiome. Nature.

[46] Lozupone C.A., Hamady M., Kelley S.T., Knight R. (2007). Quantitative and Qualitative β Diversity Measures Lead to Different Insights into Factors That Structure Microbial Communities. Appl. Environ. Microbiol..

[47] Nadal I., Santacruz A., Marcos A., Warnberg J., Garagorri M., Moreno L.A., Martin-Matillas M., Campoy C., Martí A., Moleres A. (2008). Shifts in Clostridia, Bacteroides and Immunoglobulin-Coating Fecal Bacteria Associated with Weight Loss in Obese Adolescents. Int. J. Obes..

[48] Santacruz A., Marcos A., Wärnberg J., Martí A., Martin-Matillas M., Campoy C., Moreno L.A., Veiga O., Redondo-Figuero C., Garagorri J.M. (2009). Interplay Between Weight Loss and Gut Microbiota Composition in Overweight Adolescents. Obesity.

[49] Simões C.D., Maukonen J., Scott K.P., Virtanen K.A., Pietiläinen K.H., Saarela M. (2014). Impact of a Very Low-Energy Diet on the Fecal Microbiota of Obese Individuals. Eur. J. Nutr..

[50] Louis S., Tappu R.-M., Damms-Machado A., Huson D.H., Bischoff S.C. (2016). Characterization of the Gut Microbial Community of Obese Patients Following a Weight-Loss Intervention Using Whole Metagenome Shotgun Sequencing. PLoS ONE.

[51] Frost F., Storck L.J., Kacprowski T., Gärtner S., Rühlemann M., Bang C., Franke A., Völker U., Aghdassi A.A., Steveling A. (2019). A Structured Weight Loss Program Increases Gut Microbiota Phylogenetic Diversity and Reduces Levels of Collinsella in Obese Type 2 Diabetics: A Pilot Study. PLoS ONE.

[52] Walker A.W., Ince J., Duncan S.H., Webster L.M., Holtrop G., Ze X., Brown D., Stares M.D., Scott P., Bergerat A. (2011). Dominant and Diet-Responsive Groups of Bacteria within the Human Colonic Microbiota. ISME J..

[53] Kootte R.S., Levin E., Salojärvi J., Smits L.P., Hartstra A.V., Udayappan S.D., Hermes G., Bouter K.E., Koopen A.M., Holst J.J. (2017). Improvement of Insulin Sensitivity after Lean Donor Feces in Metabolic Syndrome Is Driven by Baseline Intestinal Microbiota Composition. Cell Metab..

[54] Nirmalkar K., Murugesan S., Pizano-Zárate M.L., Villalobos-Flores L.E., García-González C., Morales-Hernández R.M., Nuñez-Hernández J.A., Hernández-Quiroz F., Del Socorro Romero-Figueroa M., Hernández-Guerrero C. (2018). Gut Microbiota and Endothelial Dysfunction Markers in Obese Mexican Children and Adolescents. Nutrients.

[55] Liu H., Zhang H., Wang X., Yu X., Hu C., Zhang X. (2018). The Family Coriobacteriaceae Is a Potential Contributor to the Beneficial Effects of Roux-En-Y Gastric Bypass on Type 2 Diabetes. Surg. Obes. Relat. Dis..

[56] Corrêa T.A.F., Rogero M.M., Hassimotto N.M.A., Lajolo F.M. (2019). The Two-Way Polyphenols-Microbiota Interactions and Their Effects on Obesity and Related Metabolic Diseases. Front. Nutr..

[57] Chávez-Carbajal A., Nirmalkar K., Pérez-Lizaur A., Hernández-Quiroz F., Ramírez-del-Alto S., García-Mena J., Hernández-Guerrero C. (2019). Gut Microbiota and Predicted Metabolic Pathways in a Sample of Mexican Women Affected by Obesity and Obesity Plus Metabolic Syndrome. Int. J. Mol. Sci..

[58] Kasai C., Sugimoto K., Moritani I., Tanaka J., Oya Y., Inoue H., Tameda M., Shiraki K., Ito M., Takei Y. (2015). Comparison of the Gut Microbiota Composition between Obese and Non-Obese Individuals in a Japanese Population, as Analyzed by Terminal Restriction Fragment Length Polymorphism and next-Generation Sequencing. BMC Gastroenterol..

[59] Liu R., Hong J., Xu X., Feng Q., Zhang D., Gu Y., Shi J., Zhao S., Liu W., Wang X. (2017). Gut Microbiome and Serum Metabolome Alterations in Obesity and after Weight-Loss Intervention. Nat. Med..

[60] Damms-Machado A., Mitra S., Schollenberger A.E., Kramer K.M., Meile T., Königsrainer A., Huson D.H., Bischoff S.C. (2015). Effects of Surgical and Dietary Weight Loss Therapy for Obesity on Gut Microbiota Composition and Nutrient Absorption. BioMed Research International.

[61] Naderpoor N., Mousa A., Gomez-Arango L.F., Barrett H.L., Dekker Nitert M., de Courten B. (2019). Faecal Microbiota Are Related to Insulin Sensitivity and Secretion in Overweight or Obese Adults. J. Clin. Med..

[62] Menni C., Zhu J., Le Roy C.I., Mompeo O., Young K., Rebholz C.M., Selvin E., North K.E., Mohney R.P., Bell J.T. (2020). Serum Metabolites Reflecting Gut Microbiome Alpha Diversity Predict Type 2 Diabetes. Gut Microbes.

[63] Monga Kravetz A., Testerman T., Galuppo B., Graf J., Pierpont B., Siebel S., Feinn R., Santoro N. (2020). Effect of Gut Microbiota and PNPLA3 Rs738409 Variant on Nonalcoholic Fatty Liver Disease (NAFLD) in Obese Youth. J. Clin. Endocrinol. Metab..

[64] Sze M.A., Schloss P.D. (2016). Looking for a Signal in the Noise: Revisiting Obesity and the Microbiome. mBio.

[65] Le Chatelier E., Nielsen T., Qin J., Prifti E., Hildebrand F., Falony G., Almeida M., Arumugam M., Batto J.-M., Kennedy S. (2013). Richness of Human Gut Microbiome Correlates with Metabolic Markers. Nature.

[66] Stanislawski M.A., Dabelea D., Lange L.A., Wagner B.D., Lozupone C.A. (2019). Gut Microbiota Phenotypes of Obesity. NPJ Biofilms Microbiomes.

[67] Yu E.W., Gao L., Stastka P., Cheney M.C., Mahabamunuge J., Soto M.T., Ford C.B., Bryant J.A., Henn M.R., Hohmann E.L. (2020). Fecal Microbiota Transplantation for the Improvement of Metabolism in Obesity: The FMT-TRIM Double-Blind Placebo-Controlled Pilot Trial. PLoS Med..

[68] De Cabo R., Mattson M.P. (2019). Effects of Intermittent Fasting on Health, Aging, and Disease. N. Engl. J. Med..

[69] Francesco A.D., Germanio C.D., Bernier M., de Cabo R. (2018). A Time to Fast. Science.

[70] Catenacci V.A., Pan Z., Ostendorf D., Brannon S., Gozansky W.S., Mattson M.P., Martin B., MacLean P.S., Melanson E.L., Troy Donahoo W. (2016). A Randomized Pilot Study Comparing Zero-Calorie Alternate-Day Fasting to Daily Caloric Restriction in Adults with Obesity. Obesity.

[71] Mattson M.P., Longo V.D., Harvie M. (2017). Impact of Intermittent Fasting on Health and Disease Processes. Ageing Res. Rev..

[72] Razavi R., Parvaresh A., Abbasi B., Yaghoobloo K., Hassanzadeh A., Mohammadifard N., Clark C.C.T., Morteza Safavi S. (2020). The Alternate-Day Fasting Diet Is a More Effective Approach than a Calorie Restriction Diet on Weight Loss and Hs-CRP Levels. Int. J. Vitam. Nutr. Res..

[73] Dao M.C., Everard A., Aron-Wisnewsky J., Sokolovska N., Prifti E., Verger E.O., Kayser B.D., Levenez F., Chilloux J., Hoyles L. (2016). Akkermansia Muciniphila and Improved Metabolic Health during a Dietary Intervention in Obesity: Relationship with Gut Microbiome Richness and Ecology. Gut.

[74] Everard A., Belzer C., Geurts L., Ouwerkerk J.P., Druart C., Bindels L.B., Guiot Y., Derrien M., Muccioli G.G., Delzenne N.M. (2013). Cross-Talk between Akkermansia Muciniphila and Intestinal Epithelium Controls Diet-Induced Obesity. Proc. Natl. Acad. Sci. USA.

[75] Shin N.-R., Lee J.-C., Lee H.-Y., Kim M.-S., Whon T.W., Lee M.-S., Bae J.-W. (2014). An Increase in the Akkermansia Spp. Population Induced by Metformin Treatment Improves Glucose Homeostasis in Diet-Induced Obese Mice. Gut.

[76] Peres Valgas da Silva C., Hernández-Saavedra D., White J.D., Stanford K.I. (2019). Cold and Exercise: Therapeutic Tools to Activate Brown Adipose Tissue and Combat Obesity. Biology.

[77] Gao X., Xie Q., Kong P., Liu L., Sun S., Xiong B., Huang B., Yan L., Sheng J., Xiang H. (2017). Polyphenol- and Caffeine-Rich Postfermented Pu-Erh Tea Improves Diet-Induced Metabolic Syndrome by Remodeling Intestinal Homeostasis in Mice. Infect. Immun..

[78] Burcelin R. (2016). Gut Microbiota and Immune Crosstalk in Metabolic Disease. Mol. Metab..

[79] Frank D.N., Zhu W., Sartor R.B., Li E. (2011). Investigating the Biological and Clinical Significance of Human Dysbioses. Trends Microbiol..

[80] Sonnenburg J.L., Bäckhed F. (2016). Diet–Microbiota Interactions as Moderators of Human Metabolism. Nature.

[81] Fabbiano S., Suárez-Zamorano N., Chevalier C., Lazarević V., Kieser S., Rigo D., Leo S., Veyrat-Durebex C., Gaïa N., Maresca M. (2018). Functional Gut Microbiota Remodeling Contributes to the Caloric Restriction-Induced Metabolic Improvements. Cell Metab..

[82] Shahar R.T., Koren O., Matarasso S., Shochat T., Magzal F., Agmon M. (2020). Attributes of Physical Activity and Gut Microbiome in Adults: A Systematic Review. Int. J. Sports Med..

[83] Mahmud R., Shehreen S., Shahriar S., Rahman M.S., Akhteruzzaman S., Sajib A.A. (2019). Non-Caloric Artificial Sweeteners Modulate the Expression of Key Metabolic Genes in the Omnipresent Gut Microbe *Escherichia Coli*. J. Mol. Microbiol. Biotechnol..

[84] Voigt R.M., Forsyth C.B., Green S.J., Engen P.A., Keshavarzian A., Cryan J.F., Clarke G. (2016). Circadian Rhythm and the Gut Microbiome. International Review of Neurobiology.

[85] Dhurandhar N.V., Schoeller D., Brown A.W., Heymsfield S.B., Thomas D., Sørensen T.I.A., Speakman J.R., Jeansonne M., Allison D.B. (2015). Energy Balance Measurement: When Something Is Not Better than Nothing. Int. J. Obes..

[86] Zeevi D., Korem T., Zmora N., Israeli D., Rothschild D., Weinberger A., Ben-Yacov O., Lador D., Avnit-Sagi T., Lotan-Pompan M. (2015). Personalized Nutrition by Prediction of Glycemic Responses. Cell.

[87] Joyce S.A., Gahan C.G.M. (2016). Bile Acid Modifications at the Microbe-Host Interface: Potential for Nutraceutical and Pharmaceutical Interventions in Host Health. Annu. Rev. Food Sci. Technol..

