# Supplementary material for: The Gut Microbiota during a Behavioral Weight Loss Intervention
Source: Nutrients. 2021 Sep 18;13(9):3248. doi: 10.3390/nu13093248 (PMC8471894; doi:10.3390/nu13093248)

Supplementary Material

Table S1. Clinical characteristics of study cohort by intervention group. There were no significant differences in clinical characteristics between the intervention groups at baseline.

	Overall	DCR	IMF	p-value
N	56	25	31	
Weight (kgs)	94.4 (16.0)	94.4 (17.0)	94.3 (15.5)	0.995
BMI	33.1 (4.4)	32.9 (4.7)	33.2 (4.1)	0.803
Waist circumference	108.5 (10.8)	109.5 (11.5)	107.6 (10.3)	0.519
Metabolic Syndrome Score	2.3 (1.0)	2.2 (1.1)	2.4 (1.0)	0.516
Metabolic Syndrome	21 (37.5)	9 (36.0)	12 (38.7)	>0.99
Triglycerides (mg/dL)	146.1 (89.0)	129.0 (64.5)	160.0 (103.6)	0.198
Glucose (mg/dL)	93.6 (9.8)	92.3 (10.3)	94.7 (9.4)	0.363
Diastolic blood pressure (mmHg)	74.2 (8.3)	73.8 (9.5)	74.6 (7.3)	0.694
Systolic blood pressure (mmHg)	118.1 (14.0)	115.5 (14.5)	120.2 (13.4)	0.218
HDL (mg/dL)	49.9 (13.5)	48.7 (12.6)	50.9 (14.3)	0.552

Table S2. Association between baseline and change in participant gut microbiota composition and two clinical outcome measures, percent change in weight and in waist circumference. The table shows p-values from Microbiome-based Kernel Association Tests (MiRKAT) using kernels based on the weighted and unweighted UniFrac metrics in the overall cohort, as well as stratified by intervention group.

DCR: Daily caloric restriction; IMF: intermittent fasting

			Clinical Outcome	
**Time**	**Participants**	**UniFrac Metric**	**Δ Weight (%)**	**Δ Waist circumference (%)**
Baseline	All	Weighted	0.277	**0.011**
		Unweighted	0.586	0.740
	DCR	Weighted	0.065	0.495
		Unweighted	0.828	0.808
	IMF	Weighted	0.299	**0.028**
		Unweighted	0.473	0.830
Change (baseline to 3 months)	All	Weighted	0.481	**0.009**
		Unweighted	0.844	0.645
	DCR	Weighted	0.931	0.449
		Unweighted	0.815	0.285
	IMF	Weighted	0.208	**0.030**
		Unweighted	0.879	0.913

Table S3. Association between participant gut microbiota alpha-diversity and outcome measures.

A) This table shows the results of longitudinal linear mixed models of weight and waist circumference based on 4 indices of alpha-diversity: Observed OTUs, Evenness, Shannon diversity index, and Faith's Phylogenetic Diversity. These indices reflect different aspects of diversity, such as the richness, evenness, and phylogenetic relatedness of the organisms detected in the samples.

B) This table shows the results of linear regressions models of percent change in weight and waist circumference based on baseline indices of alpha-diversity.

DCR: Daily caloric restriction; IMF: intermittent fasting; OTUs: operational taxonomic units (clusters of organisms that are grouped by gene sequence similarity); PD: Phylogenetic Diversity.

A)

Outcome	Longitudinal Measure	Estimate	Lower CI	Upper CI	p-value
Weight (kg)	Observed OTUs	-0.01	-0.03	0.00	0.179
	Evenness	-3.12	-16.96	10.83	0.665
	Shannon	-0.74	-1.99	0.51	0.254
	Faith's PD	-0.04	-0.10	0.02	0.176
Waist circumference (cm)	Observed OTUs	-0.01	-0.03	0.02	0.516
	Evenness	-10.48	-29.73	8.97	0.297
	Shannon	-1.02	-2.76	0.74	0.261
	Faith's PD	-0.02	-0.11	0.06	0.574

B)

Outcome	Baseline Measure	Estimate	Lower CI	Upper CI	p-value
Change in weight (%)	Observed OTUs	0.00	-0.0001	0.0003	0.352
	Evenness	0.07	-0.08	0.22	0.348
	Shannon	0.01	-0.01	0.02	0.296
	Faith's PD	0.0004	-0.0004	0.001	0.296
Change in waist circumference (%)	Observed OTUs	0.0001	-0.0002	0.0004	0.675
	Evenness	0.12	-0.08	0.32	0.233
	Shannon	0.01	-0.01	0.03	0.309
	Faith's PD	0.0002	-0.0008	0.001	0.656

Table S4. Performance metrics of random forests to predict percent change in weight and in waist circumference using all taxa and those selected as most highly predictive of these outcomes by VSURF.

This table shows the model performance metrics (R^2, or the percent of the variation explained in the outcome; mean absolute error [MAE]; and root mean squared error [RMSE]) of random forests predicting the clinical outcomes based on baseline abundance of gut microbiota taxa and on change in taxa from baseline to 3 months. These results are from 3-fold cross validation of 100 repetitions.

Outcome	Time	Model	R^2 (%)	MAE (%)	RMSE
Δ Weight (%)	Baseline	Random Forests of all taxa	2.02 (0.66-5.04)	2.99 (2.78-3.21)	3.66 (3.32-4.06)
		Random Forests of VSURF selected taxa	29.36 (20.63-40.78)	2.46 (2.2-2.74)	3.16 (2.79-3.52)
	Change (baseline to 3 months)	Random Forests of all taxa	2.31 (0.59-7.33)	3.09 (2.92-3.31)	3.78 (3.46-4.15)
		Random Forests of VSURF selected taxa	21.31 (12.39-31.85)	2.7 (2.43-2.98)	3.38 (3.04-3.81)
Δ Waist circumference (%)	Baseline	Random Forests of all taxa	3.82 (0.73-10.11)	3.64 (3.35-3.94)	4.78 (4.35-5.28)
		Random Forests of VSURF selected taxa	21.25 (10.43-33.48)	3.53 (3.19-3.85)	4.39 (3.97-4.81)
	Change (baseline to 3 months)	Random Forests of all taxa	14.56 (6.93-24.54)	3.61 (3.28-3.87)	4.8 (4.38-5.16)
		Random Forests of VSURF selected taxa	37.97 (23.51-48.39)	3.32 (3.02-3.55)	4.08 (3.77-4.47)

Table S5. Regression models of the change in alpha-diversity as a function of change in weight or waist circumference, change in dietary intake and change in physical activity.

Model	N	Outcome	Alpha Diversity	Covariate (change in measure)	Estimate	Lower CI	Upper CI	P-value
Dietary	45	Weight	Evenness	Weight	-0.12	-0.82	0.58	0.73
				Kilocalories	0.000002	-0.0001	0.0001	0.95
				% Protein	0.0012	-0.01	0.009	0.75
				% Fat	0.0009	-0.005	0.007	0.77
				% Carbohydrates	-0.0008	-0.007	0.005	0.79
			Shannon	Weight	-1.53	-9.18	6.12	0.69
				Kilocalories	0.0002	-0.001	0.001	0.61
				% Protein	0.030	-0.05	0.11	0.46
				% Fat	0.01	-0.05	0.08	0.67
				% Carbohydrates	0.017	-0.05	0.08	0.60
			Faith's PD	Weight	-21.02	-183.46	141.42	0.79
				Kilocalories	0.00	-0.01	0.02	0.55
				% Protein	0.43	-1.32	2.19	0.62
				% Fat	0.11	-1.32	1.54	0.87
				% Carbohydrates	0.77	-0.62	2.17	0.27
			Observed Species	Weight	-63.62	-654.22	526.97	0.83
				Kilocalories	0.02	-0.05	0.08	0.61
				% Protein	1.93	-4.45	8.32	0.54
				% Fat	0.93	-4.26	6.12	0.72
				% Carbohydrates	3.09	-1.97	8.16	0.22
		Waist circumference	Evenness	Waist circumference	-0.11	-0.71	0.48	0.70
				Kilocalories	0.000003	-0.00007	0.0001	0.93
				% Protein	0.001	-0.01	0.009	0.79
				% Fat	0.001	-0.005	0.007	0.78
				% Carbohydrates	-0.0006	-0.007	0.005	0.85
			Shannon	Waist circumference	-1.61	-8.06	4.83	0.61
				Kilocalories	0.0002	-0.001	0.001	0.59
				% Protein	0.03	-0.06	0.11	0.50
				% Fat	0.014	-0.05	0.08	0.68
				% Carbohydrates	0.020	-0.04	0.08	0.53
			Faith's PD	Waist circumference	-37.80	-174.40	98.80	0.58
				Kilocalories	0.01	-0.01	0.02	0.53
				% Protein	0.34	-1.44	2.13	0.70
				% Fat	0.11	-1.31	1.54	0.87
				% Carbohydrates	0.82	-0.55	2.19	0.23
			Observed Species	Waist circumference	-88.98	-586.74	408.78	0.72
				Kilocalories	0.02	-0.04	0.08	0.59
				% Protein	1.75	-4.75	8.25	0.59
				% Fat	0.93	-4.25	6.11	0.72
				% Carbohydrates	3.22	-1.77	8.21	0.20
Physical Acvity	47	Weight	Evenness	Weight	-0.07	-0.74	0.60	0.83
				Stepping time	0.0005	-0.001	0.002	0.51
				Sitting time	0.000005	-0.0002	0.0002	0.96
			Shannon	Weight	-2.39	-9.70	4.91	0.51
				Stepping time	0.00	-0.01	0.02	0.61
				Sitting time	-0.0008	-0.003	0.001	0.46
			Faith's PD	Weight	-55.23	-212.65	102.19	0.48
				Stepping time	0.06	-0.27	0.39	0.72
				Sitting time	-0.03	-0.08	0.02	0.20
			Observed Species	Weight	-199.08	-758.70	360.53	0.48
				Stepping time	0.22	-0.95	1.40	0.70
				Sitting time	-0.13	-0.30	0.04	0.12
		Waist circumference	Evenness	Waist circumference	-0.08	-0.60	0.44	0.77
				Stepping time	0.0005	-0.001	0.002	0.49
				Sitting time	0.000004	-0.0002	0.0002	0.97
			Shannon	Waist circumference	-1.00	-6.69	4.69	0.72
				Stepping time	0.00	-0.01	0.02	0.52
				Sitting time	-0.0008	0.00	0.00	0.48
			Faith's PD	Waist circumference	-14.76	-137.61	108.09	0.81
				Stepping time	0.09	-0.24	0.41	0.60
				Sitting time	-0.03	-0.08	0.02	0.23
			Observed Species	Waist circumference	-43.16	-480.05	393.74	0.84
				Stepping time	0.32	-0.83	1.48	0.57
				Sitting time	-0.13	-0.29	0.04	0.14

Table S6. Association between baseline and change in participant gut microbiota composition and two clinical outcome measures, percent change in weight and in waist circumference, controlling for change in dietary intake and physical activity. The table shows p-values from Microbiome-based Kernel Association Tests (MiRKAT) using kernels based on the weighted and unweighted UniFrac metrics in the overall cohort.

Time	Model	UniFrac Metric	Clinical Outcome	
			Δ Weight (%)	Δ Waist circumference (%)
Baseline	Controlling for diet, age and sex (N=54)	Weighted	0.401	**0.011**
	Caloric intake, % carbohydrates / protein / fat	Unweighted	0.342	0.160
Change (baseline to 3 months)	Controlling for change in diet, age, sex and group (N=45)	Weighted	0.792	**0.023**
	Change in: caloric intake, % carbohydrates / protein / fat	Unweighted	0.923	0.455
Baseline	Controlling for diet, physical activity, age and sex (N=55)	Weighted	0.306	**0.002**
	≥75 cadence stepping time, sitting time	Unweighted	0.775	0.558
Change (baseline to 3 months)	Controlling for change in physical activity, age, sex and group (N=47)	Weighted	0.557	**0.027**
	Change in: ≥75 cadence stepping time, sitting time	Unweighted	0.980	0.575

Figure S1. Change in diet and physical activity of DRIFT2 participants from baseline to 3 months and the association with change in the gut microbiota community structure (beta-diversity).

(A) During the first three months of the intervention, participants decreased dietary energy intake and percentage intake from fat, and they increased percentage intake from protein.

(B) The gut microbiota may contribute towards weight loss, but it also may change in response to weight loss. We used permutational ANOVA to quantify predictors of the change from baseline to 3 months in gut microbiota beta diversity (weighted and unweighted UniFrac). The plots show predictors on the y-axes and R2 values (amount of variation explained in the change in gut microbiota beta diversity) on the x-axes. This plot show results of models including change in weight, as well change in diet (upper plots; kilocalories and percentages of macronutrients) and change in physical activity (lower plots; >75 cadence stepping time and sitting time). Sex, age and intervention group were included as control variables. The color indicates the statistical significance as indicated in the legend.

(C) This plot is similar to B, but includes change in waist circumference rather than weight.

A

	**Baseline**	**3 months**	**Change**	**P-value**
Dietary Intake N	*54*	*48*	*45*	
Dietary energy intake	1714.0 (333.2)	1275.8 (387.4)	-469.6 (435.3)	<0.001
% Fat	38.5 (6.5)	34.9 (5.8)	-3.8 (6.2)	<0.001
% Protein	17.5 (3.6)	21.2 (4.6)	3.7 (4.9)	<0.001
% Carbohydrates	41.6 (7.6)	41.5 (7.1)	-0.1 (6.2)	0.9
Physical Activity N	*55*	*50*	*47*	
Stepping time at cadence ≥75 steps/min (min/day)	49.7 (17.0)	62.2 (25.0)	12.6 (19.5)	<0.001
Sitting time (min/day)	550.1 (92.6)	518.4 (106.7)	-31.1 (124.6)	0.09

B

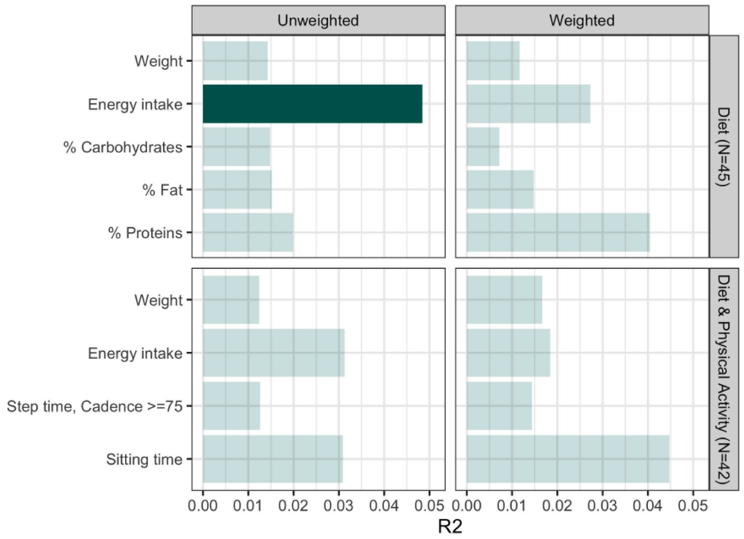

C

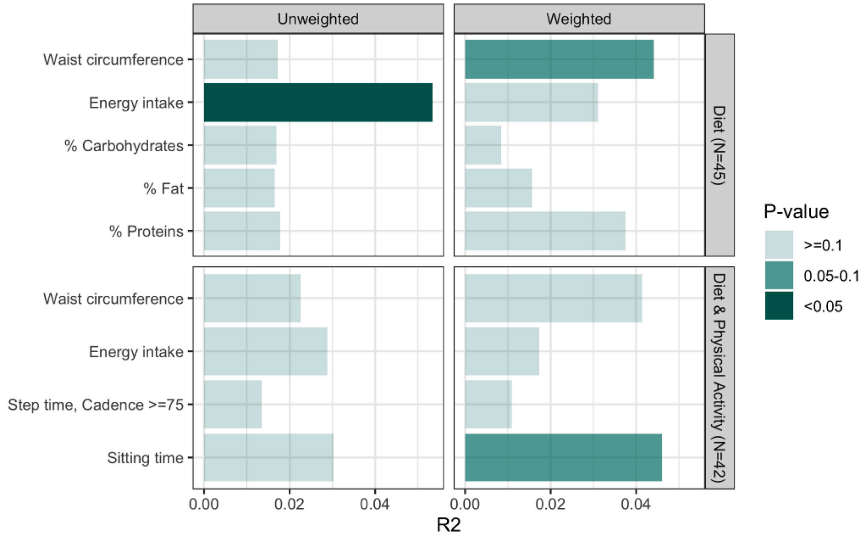

Figure S2. Changes in the gut microbiota taxa of DRIFT2 participants from baseline to 3 months by intervention group assignment. The overall gut microbiota composition of study participants changed significantly from baseline to 3 months, but there was not a statistically significant interaction between time and intervention group, suggesting that the changes in the overall gut microbiota composition were comparable for DCR and IMF.

(A) Average relative abundance of the most prevalent gut microbiota phyla among DCR and IMF at baseline and 3 months. This plot shows comparable shifts over time in the predominant phyla among the two intervention groups.

(B) Average relative abundance of the most prevalent gut microbiota genera among DCR and IMF at baseline and 3 months. As seen at the phylum-level, the intervention groups showed comparable shifts in genera. The one statistically significant exception was the genus *Akkermansia* (see Figure 5).

DCR: Daily caloric restriction; IMF: intermittent fasting

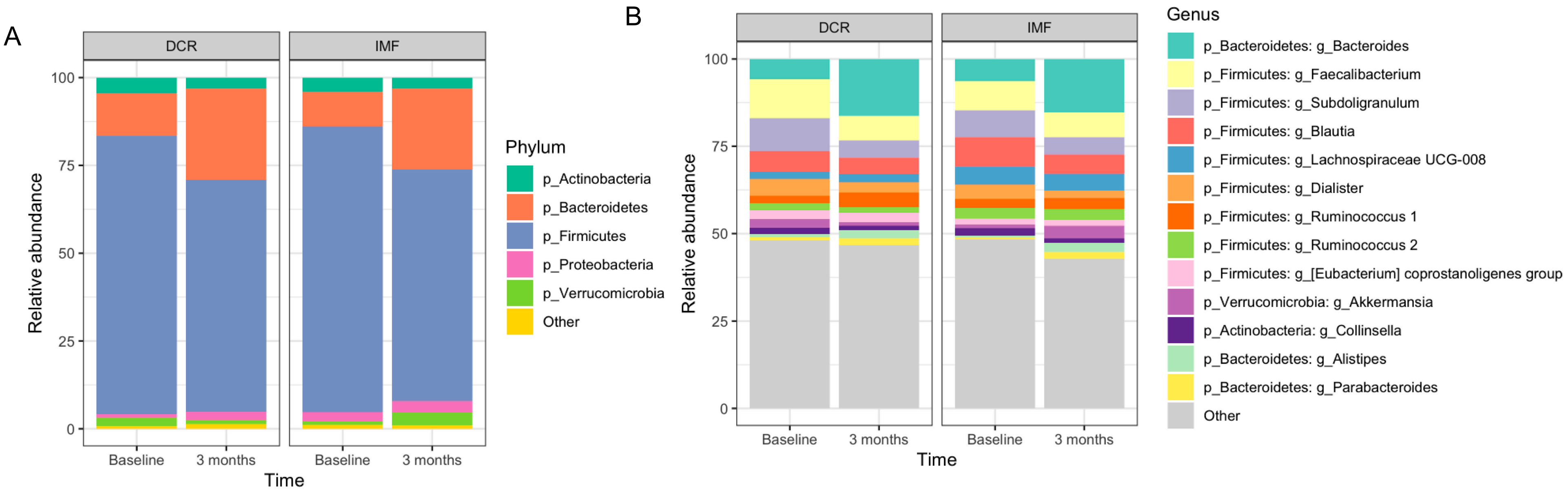

Supplement: Supplementary file 1 [file nutrients-13-03248-s001.zip › Supplement 032421.pdf]
